# Skin metastasis of BRCA mutated prostate cancer: A case report and a brief review of literature

**DOI:** 10.1097/MD.0000000000040016

**Published:** 2024-10-11

**Authors:** Salim Jubran, Umberto Basso, Anna Milani, Elisa Erbetta, Andrea Di Marco, Chiara Pittarello, Nicolò Cavasin, Eleonora Lai, Silvia Stragliotto, Francesco Pierantoni, Ilaria Zampiva, Davide Bimbatti, Marco Maruzzo

**Affiliations:** a Oncology Unit 1, Istituto Oncologico Veneto, IOV – IRCCS, Padova, Italy; b Department of Surgery, Oncology and Gastroenterology, University of Padua, Padua, Italy; c Oncology Unit 3, Istituto Oncologico Veneto, IOV – IRCCS, Padova, Italy; d Department of Engineering for Innovation Medicine (DIMI), Section of Innovation Biomedicine – Oncology Area, University of Verona, Verona, Italy.

**Keywords:** BRCA mutation, case report, castration-resistant prostate cancer, cutaneous metastasis, PARPi

## Abstract

**Rationale:**

Metastatic castration-resistant prostate cancer has a poor prognosis especially when harboring DNA damage repair gene mutations, nevertheless, in the case of pathogenic *BRCA* gene mutations, PARPi demonstrated a survival benefit and is a validated treatment. Nowadays, there is no data regarding unusual metastases after these drugs. Cutaneous metastases appear rarely in prostate cancer and were associated with a worse prognosis. Moreover, there are no consolidated data concerning skin tropism of prostate cancer cells, neither in the case of BRCA-associated cancers.

**Patient concerns:**

Here, we report the case of a patient with a long history of BRCA1-mutated metastatic castration-resistant prostate cancer who developed a skin lesion on the scalp while on his fifth line of systemic therapy with olaparib. After a complete radical surgical excision, the pathology report showed prostate cancer localization.

**Diagnoses:**

A diagnosis of skin metastasis from prostate cancer was reported.

**Outcomes:**

The patient then continued olaparib therapy; after 7 months from excision, he experienced further bone and biochemical progression but not cutaneous progression.

**Lessons:**

A literature review of all reported cases of cutaneous metastasis in prostate cancer was conducted to shed light on the incidence, clinical presentation, diagnosis, treatment, and prognosis of this entity. We also reviewed published cases of skin metastasis in BRCA-associated cancers with an effort to correlate skin involvement with PARPi treatment, BRCAness status, and prognosis.

## 1. Introduction

Metastatic castration-resistant prostate cancer (mCRPC) is a heterogeneous disease with poor prognosis^[1]^ with median survival of 36 to 42 months.^[2]^ Systemic therapeutic options for mCRPC vary, including new endocrine drugs, chemotherapy, molecular-target therapy, bone-targeted therapy, radioligand treatment and other investigational drugs still under research.^[3]^

For instance, abiraterone with prednisolone combined with androgen deprivation therapy (ADT) should be nowadays considered a new standard treatment for patients with high-risk nonmetastatic prostate cancer, while in metastatic setting, enzalutamide and abiraterone should not be combined for those starting long-term ADT^[4]^

Approximately one/third of patients harbor deleterious aberrations in genes involved in DNA damage repair (DDR), specifically homologous recombination repair (HRR) pathway^[5]^: 6 primary pathways of DDR have been identified, which are variably used to address double-strand DNA breaks (DSB) and single-strand DNA breaks (SSB) damage from a variety of mechanisms of injury. Homologous recombination (HR) and nonhomologous end joining (NHEJ) recombination are the 2 major pathways responsible for repairing DSB.^[6]^ Moreover, the genes most frequently involved in prostate cancer belong to breast cancer (BRCA) family genes, either acquired in the tumor tissue or germline and inheritable. The incidence of somatic and germline BRCA mutations is 13% and 6%, respectively (BRCA1 represents the minority of cases).^[7–9]^ In this context, a unique genome instability phenotype is represented by biallelic inactivation of CDK12. The CDK12-specific focal tandem duplications can lead to the differential expression of oncogenic drivers, such as CCND1 and CDK4. As such, there is a possibility of vulnerability to CDK4/6 inhibitors for CDK12-mutated tumors. Moreover, the CDK12 aberrations may be used next to mismatch repair deficiency, as a biomarker of treatment response. This highlights the rationale for the combination therapeutic strategy of immune checkpoint blockade and CDK4/6 inhibition also in prostate cancer, even if up to date clinical results with these drugs are very poor.^[10]^

In prostate cancer, BRCA gene mutations are associated with unfavorable clinicopathological features such as younger age at diagnosis, higher Gleason score (GS), nodal involvement, metastatic disease at diagnosis, increased risk of relapse and shorter time of development of castration resistance as well as reduced progression free survival (PFS) and overall survival (OS), and should therefore be considered as a negative prognostic marker;^[11]^ these gene alterations have also a therapeutic value, predicting through synthetic lethality mechanism the response to poly adenosine diphosphate ribose polymerase inhibitors (PARPi).^[7–10]^ Specifically, PARP-DNA complexes have the ability to interfere with DNA replication, and it has been indicated that PARP trapping is important for the cytotoxicity of PARPi. This explains the different magnitude of cytotoxicity exerted by different PARPi. Among PARPi that have already been evaluated, olaparib, niraparib, and rucaparib trap PARP approximately 100-fold more efficiently than veliparib.^[12]^

PROfound study – a randomized phase III, open label, mutation-driven trial – was conducted to assess the potential benefits of olaparib in patients with mCRPC after disease progression on prior androgen receptor targeting agent. The patients were divided in 2 cohorts: cohort A included patients with BRCA1, BRCA2, and ATM alterations, and cohort B included patients with other HRR pathway alterations. Olaparib showed radiographic PFS benefits both in cohort A (HR 0.34, 95% CI: 0.25–0.47, *P* < .001) and in the whole population (HR 0.49, 95% CI: 0.38–0.63, *P* < .001). OS was also improved by olaparib in cohort A (19.1 vs 14.7 months; HR 0.69, 95% CI: 0.50–0.97). In the overall population a trend of improved OS was also noted (median OS 17.3 vs 14 months; HR 0.79, 95% CI: 0.61–1.03).^[1,13,14]^

Metastatic prostate cancer usually involves bones (about 85%), distant lymph nodes, liver and lung (respectively about 10% each site) and rarely other sites like brain, adrenal gland and gastrointestinal tract (about 3% each site).^[15]^ Nevertheless, very rare cutaneous metastases may occasionally appear with a reported rate <1%.^[16–18]^

In this case report, we present a patient with BRCA1 mutated mCRPC who developed oligo-progressive skin metastasis to the scalp, successfully managed with excision, and discuss literature data concerning skin metastases of prostate cancer and BRCA-associated cancers.

## 2. Case presentation

A 64-year-old Caucasian male was known to our medical oncology unit for his long history of prostate cancer. He also suffered from well controlled hypertension and reported a positive familial history of prostate cancer. In 2010, he presented with hematuria and swelling of the laterocervical lymph nodes; blood tests detected a prostate specific antigen (PSA) value of 90 ng/mL and a total body computed tomography scan showed bladder infiltration from the prostate gland and abdominal and lateral cervical lymphadenopathies. Two biopsies were performed, on the left laterocervical lymph node and on the prostatic lesion. They both resulted positive for poorly differentiated prostate carcinoma (GS 10), stage IV cT4N1M1. The patient received complete androgen blockade therapy followed by systemic chemotherapy with carboplatin plus docetaxel, achieving biochemical and radiological response. Three years later, he received systemic treatment with docetaxel plus prednisone (10 cycles) for mCRPC setting along with zoledronic acid for bone disease progression. In February 2017 for biochemical and radiological bone progression with partial cauda equina syndrome (numbness on feet and sacral pain), the patient received enzalutamide and underwent radiotherapy treatment on the left sacral region. The patient maintained the same treatment with biochemical, clinical and radiological response for 4.5 years with PSA nadir 0.17 ng/mL in June 2018. At this point, DDR deficiency was assessed using the Foundation Medicine gene mutation panel on the tumoral tissue (FoundationOne). A class 5 pathogenic variant was detected in the BRCA1 gene (L1764fs*1; truncating oncogenic mutation); germline analysis was negative for the same mutation. In August 2021, due to bone progression with PSA 20 ng/mL, the patient started treatment with cabazitaxel (8 cycles) associated with the intensification of zoledronic acid infusions with partial biochemical response and radiological stability at subsequent CT scans until January 2023, when he further experienced asymptomatic biochemical (PSA 17.3 ng/mL) and radiographic progression on the known bone lesions. He therefore started olaparib with radiological and clinical stability but no biochemical control and, 4 months later, asymptomatic pulmonary embolism was detected. Olaparib dosage was reduced by 2 levels due to anemia grade 3 and, in October 2023, the patient underwent radiotherapy on the right scapula due to pain. In November 2023, the patient reported the onset of a small new nodule on his scalp, PSA was 43.78 ng/mL. At physical examination, the lesion appeared as a nodule of around 10 millimeters in diameter, dyschromic, slightly elevated and ulcerated, with irregular borders, without itching. After plastic surgery consultation, the lesion was totally excised. The histological report revealed malignant infiltration of the dermo-epidermal tissue with nodular solid growth pattern and residual acinar structures; margins were negative and the immunohistochemical profile resulted compatible with prostatic neoplasm (NKX3+, PSA+, PSAP+, S100−, SOX10−, MART1−, CK7−, p63−, p40−, GATA3−).

Currently, he continuing treatment with olaparib 200 mg bid, well tolerated. The last radiological assessment, conducted in April 2024, showed further bone oligo-progression to the right femur thus the patient is under evaluation for stereotactic radiation therapy. He is doing well and he is satisfied with the treatments received so far.

## 3. Discussion

The skin is a metastatic site for primary solid cancers with a reported incidence that ranges from 0.9% to 9%, with breast cancer having the highest occurrence rate, and prostate cancer the lowest.^[16]^ For prostate cancer, Mueller et al reported an incidence of 0.36% and a recent literature review reported an incidence of 0.62%; skin metastasis appears late in the disease course and is associated with poor prognosis.^[17–19]^

Cutaneous neoplastic spread from prostate cancer can manifest with different possible patterns: single or multiple papulonodular lesions, inflammatory erysipeloid plaques, or sclerodermoid lesions. Most frequently, they appear as multiple nodules or round plaques from several millimeters to centimeters with color from flesh, to erythematous or violaceous, and rarely like a nonspecific macular rash or telangiectatic lesion.^[19–21]^ They can be symptomatic for itching, ulceration, pain or bleeding. Most common sites are the inguino-abdomino-perineal region (51%), thoracic wall and back (23%), neck and head (16%) and extremities (10%). Rare cases have been also reported in other sites.^[22–27]^

Diagnosis is often made in advanced stage of the disease, and skin lesions are rarely the first manifestation of prostate cancer.^[28,29]^ During assessment of a new skin eruption in cancer patients, several possibilities should be considered in the differential diagnosis, besides the uncommon occurrence of metastases: drug reactions, opportunistic infections, pyogenic granuloma, amelanotic melanoma, clear cell acanthoma, basal cell carcinoma, herpes zoster, telangiectasia, sebaceous cysts, morphea and trichoepitheliomas, among others.^[30]^ The lesion predominantly involves the dermis and rarely extends to epidermis, so shave biopsy is discouraged.^[17,19,31]^ From a histopathologic point of view, gland forming structures support the diagnosis of adenocarcinoma and vascular invasion supports the diagnosis of metastatic lesion. Direct histologic comparison with the primary tumor may be difficult due to undifferentiated histology. The use of immunohistochemical (IHC) stains can confirm whether the lesion is a primary or secondary tumor. The specific IHC markers for prostate cancer are NKX3.1, PSA, PSAP, PSMA, and P501S.^[32–34]^ Furthermore, negative PSA does not exclude the prostatic origin especially in the case of undifferentiated tumors.^[22]^ Finally, the expression of human Ki-67 protein and CD10 can also help to confirm suspected metastatic lesions.^[35,36]^

The treatment is primarily palliative and based on clinical presentation, skin localizations and patient preference in an attempt to improve associated symptoms and quality of life, mostly in presence of psychosocial distress due to visible or odorous lesions. Options can vary from ablative radiotherapy to electrochemotherapy, systemic anticancer treatment or surgery excision. Other supportive therapies include topical or systemic procoagulants for bleeding, optimal local wound care in case of ulceration, infection or crusting lesions, with application of topical medications or debridement if needed, and antalgic drugs for pain relief.^[29,37]^ Systemic treatment should be considered in case of progressive disease with multiple cutaneous metastases; there are reported cases with response to radiotherapy^[33,38]^ and also to androgen receptor signaling inhibitors.^[28]^ In 1 case, docetaxel showed a complete response.^[18]^ Surgery excision, with subsequent bleeding that was treated successfully with radiotherapy, has been also reported.^[29]^ One recent case report showed a response to 177Lu-PSMA-617 radioligand therapy.^[39]^ It is notable that treatment of the primary malignancy results in partial improvement of cutaneous metastases in 65% of cases within a period of 4 to 8 weeks of start of treatment.^[27]^ One case reported a somatic CHEK2 mutation that had progressed on hormonal therapy.^[40]^ Indeed, advanced systemic involvement in the onset of skin metastases indicates a poorer prognosis,^[25,41]^ although some patients have survived for more than 3 years.^[42,43]^

Currently, there are a hundred case reports of cutaneous metastasis from prostate cancer described in literature, this number is expected to increase due to new drugs prolonging survival, greater attention to this phenomenon and diagnostic techniques.^[44]^ Furthermore, no consolidated data is available regarding the correlation of skin metastasis with BRCA gene mutated cancers even in its highest occurrence rate in breast cancer. Table 1 summarizes the published cases of BRCA-positive cancers with skin metastases.

**Table 1:** Case reports of BRCA-positive cancers with skin metastases.

Case report	Cancer type and stage*	Sex	Age†	Molecular mutation	Disease sites	Systemic treatment lines‡	Skin presentation and onset	Local skin therapy	Skin disease outcome	Outcome$
Rhiem et al, 2009	Synchronous bilateral breast cancer: stage IA (MC, TNBC) + stage IIA (ILC, TNBC)	F	35	gBRCA1	Brain, lymph nodes, skin, pleural effusion	Cisplatin-etoposide; trastuzumab maintenance; docetaxel-trastuzumab; cisplatin-gemcitabine	OPD with skin metastasis‖ of the breast, 3 yr after first diagnosis and on 2nd-line therapy	No	PR on 3rd-line (PFS 6 mo)	44
Caputo et al, 2023	Metachronous bilateral breast cancer: stage IIB (IDC, TNBC) + stage IA (TNBC)	F	38	gBRCA1	Skin, lymph nodes, bone	Paclitaxel; carboplatin-LAG525; olaparib	ORD with skin metastasis of left breast skin, 7 yr after first diagnosis and 2 yr from second surgery	No	PR on 2nd-line (PFS 14 mo); CR on 3rd-line (PFS > 40 mo)	ND
Minick et al, 2023	Breast cancer: stage ND (IDC, luminal B)	F	NS	sBRCA2 (germline negative)	Skin, bone	Palbociclib-fulvestrant; everolimus-letrozole; olaparib	ORD with skin metastasis of right upper arm on adjuvant hormonal therapy	Excision plus RT	CR on all lines; olaparib PFS > 24 mo	ND
Schwartzberg and Kiedrowski, 2021	Breast cancer stage III (ILC luminal B)	F	57	sBRCA2 (germline negative)	Bone, skin, liver, pleural effusion	CMF regimen; everolimus-exemestane; eribulin; palbociclib-fulvestrant; capecitabine; olaparib; vinorelbine; doxorubicin; carboplatin	OPD with skin metastases of left chest and back, 4 yr after first diagnosis and on 3rd-line therapy	RT plus carboplatin for ulcerated skin lesions	CR after 5th-line (PFS 6 mo); PR on 6th-line (PFS 10 mo); PD on 7th-line; PR on 8th-line (PFS 3 mo)	96
Oh et al, 2017	Ovarian seromucinous carcinoma stage IIIC	F	44	gBRCA1	Peritoneal carcinosis, adrenals, psoas muscle, lymph nodes, skin	1st/2nd/3rd lines NS; cisplatin-cyclophosphamide-doxorubicin; docetaxel-carboplatin	OPD with skin metastases of left flank and upper left arm on 3^rd^-line therapy	No	PD after 5th-line (PFS 2 mo)	60
Nie et al, 2022	Ovarian serous adenocarcinoma stage IIIC	F	62	BRCA1 (NS if somatic or germline)	Skin, lymph nodes	Carboplatin-paclitaxel with subsequent olaparib maintenance	Synchronous skin metastasis to umbilicus at first diagnosis	Excision (interval debulking surgery)	CR on olaparib maintenance	ND
Assaf et al, 2021	Pancreatic adenocarcinoma stage IIB	M	54	gBRCA2	Liver, lung, lymph nodes, skin	FOLFIRINOX regimen; cisplatin-fluorouracil with subsequent olaparib maintenance	OPD with skin metastases of face and scalp, 3 yr after first diagnosis and 4 mo after olaparib maintenance therapy	No	PD on olaparib maintenance	36
Dills et al, 2021	Prostate adenocarcinoma GS 9 stage IVB	M	64	sBRCA2 inactivation (germline NS)	Bone, lymph nodes, liver, skin	Androgen deprivation therapy; abiraterone-prednisone; docetaxel; cabazitaxel	OPD with skin metastases of inguino-abdominal area, 3 yr after first diagnosis and 1 mo after abiraterone 1st-line therapy	No	CR on 3rd-line (PFS 5 mo)	ND (patient was candidate to BSC)
Present case	Prostate adenocarcinoma GS 10 stage IVB	M	54	sBRCA1 (germline negative)	Bone, lymph nodes, skin	Carboplatin-docetaxel; docetaxel; enzalutamide; cabazitaxel; olaparib	OPD with skin metastasis of scalp, 13 yr after first diagnosis and 10 mo after olaparib 5th-line therapy	Excision	CR on 5th-line (PFS not reached)	ND (olaparib ongoing)

Abbreviations: BSC = best supportive care, CR = complete response, gBRCA = germline mutation, GS = Gleason score, IDC = invasive ductal carcinoma, ILC = invasive lobular carcinoma, MC = medullary carcinoma, ND = not determined, NS = not specified, OPD = oligo-progressive disease, ORD = oligo-recurrence disease, PD = progressive disease, PFS = progression free survival, PR = partial response, RT = radiotherapy, sBRCA = somatic mutation, TNBC = triple negative breast cancer.

* At first diagnosis.

† At metastatic stage.

‡ In chronological order.

$ Survival in months.

‖ Not like all other cases, this group did not provide a histological confirmation of skin lesions.

Regarding prostate cancer, we found only 1 case report with BRCA2 inactivation; skin disease response to docetaxel and subsequent liver involvement as a final progression of the disease were also described.^[30]^

Some cases of skin metastases in other BRCA mutated neoplasms have been reported. One case reported a poor prognosis in a 45-year-old patient with BRCA1 mutated ovarian cancer.^[45]^ A second case showed a 62-year-old patient with umbilical metastasis as the first presentation of ovarian serous adenocarcinoma with BRCA1 mutation.^[46]^

Concerning BRCA mutation-related breast cancers, several case reports have described skin metastases in BRCA-related neoplasms, mostly in triple negative breast cancer and most of them achieving long lasting response with PARPi and/or platinum-based chemotherapy.^[47,48]^

Moreover, 1 case of metastatic BRCA-related pancreatic adenocarcinoma, with distant skin lesions, has been reported with ominous prognosis.^[49]^

In the present case report, our patient had developed skin metastasis after 10 months of olaparib as a fifth-line therapy. It remains difficult to establish whether this rare metastasis is related to clonal selection induced by PARPi therapeutic pressure or to an intrinsic skin metastatic potential of BRCA status. What is certain is that this new life-prolonging drug does permit the development of rare sites of metastases. Further case reports and retrospective studies harboring BRCA mutation with unusual metastasis should be encouraged alongside preclinical and clinical research.

## 4. Conclusions

In this paper, we reported, to the best of our knowledge, the first case of prostate cancer with cutaneous metastasis to the scalp owing to somatic BRCA1 mutation. We encourage periodic physical examination and diagnostic biopsy if skin lesion is suspected during follow up visits for systemic therapy and, therefore, we strongly recommend BRCA gene analysis in the metastatic setting or earlier if familial history is present.

## Author contributions

**Conceptualization:** Salim Jubran, Umberto Basso, Francesco Pierantoni, Davide Bimbatti, Marco Maruzzo.

**Data curation:** Salim Jubran, Anna Milani, Elisa Erbetta, Chiara Pittarello, Eleonora Lai.

**Formal analysis:** Elisa Erbetta.

**Investigation:** Andrea Di Marco, Chiara Pittarello, Nicolò Cavasin.

**Methodology:** Nicolò Cavasin, Marco Maruzzo.

**Supervision:** Umberto Basso, Davide Bimbatti, Marco Maruzzo.

**Validation:** Eleonora Lai, Silvia Stragliotto, Francesco Pierantoni, Ilaria Zampiva, Davide Bimbatti, Marco Maruzzo.

**Visualization:** Umberto Basso, Elisa Erbetta, Silvia Stragliotto, Davide Bimbatti.

**Writing – original draft:** Salim Jubran, Anna Milani, Andrea Di Marco.

**Writing – review & editing:** Ilaria Zampiva, Marco Maruzzo.

## References

[1] de Bono J Mateo J Fizazi K. Olaparib for metastatic castration-resistant prostate cancer. N Engl J Med. 2020;382:2091–102. 32343890 10.1056/NEJMoa1911440

[2] Saad F Clarke NW Oya M. Olaparib plus abiraterone versus placebo plus abiraterone in metastatic castration-resistant prostate cancer (PROpel): final prespecified overall survival results of a randomised, double-blind, phase 3 trial. Lancet Oncol. 2023;24:1094–108. 37714168 10.1016/S1470-2045(23)00382-0

[3] Cai M Song XL Li XA. Current therapy and drug resistance in metastatic castration-resistant prostate cancer. Drug Resist Updat. 2023;68:100962. 37068396 10.1016/j.drup.2023.100962

[4] Attard G Murphy L Clarke NW. STAMPEDE investigators. Abiraterone acetate plus prednisolone with or without enzalutamide for patients with metastatic prostate cancer starting androgen deprivation therapy: final results from two randomised phase 3 trials of the STAMPEDE platform protocol. Lancet Oncol. 2023;24:443–56. 37142371 10.1016/S1470-2045(23)00148-1 PMC7616864

[5] Armenia J Wankowicz SAM Liu D. PCF/SU2C International Prostate Cancer Dream Team. The long tail of oncogenic drivers in prostate cancer. Nat Genet. 2018;50:645–51. 29610475 10.1038/s41588-018-0078-z PMC6107367

[6] Aliyuda F Moschetta M Ghose A. Advances in ovarian cancer treatment beyond PARP inhibitors. Curr Cancer Drug Targets. 2023;23:433–46. 36757037 10.2174/1568009623666230209121732

[7] Castro E Goh C Olmos D. Germline BRCA mutations are associated with higher risk of nodal involvement, distant metastasis, and poor survival outcomes in prostate cancer. J Clin Oncol. 2013;31:1748–57. 23569316 10.1200/JCO.2012.43.1882 PMC3641696

[8] Messina C Cattrini C Soldato D. BRCA mutations in prostate cancer: prognostic and predictive implications. J Oncol. 2020;2020:4986365. 32963528 10.1155/2020/4986365 PMC7492871

[9] Lukashchuk N Barnicle A Adelman CA. Impact of DNA damage repair alterations on prostate cancer progression and metastasis. Front Oncol. 2023;13:1162644. 37434977 10.3389/fonc.2023.1162644 PMC10331135

[10] Boussios S Rassy E Shah S Ioannidou E Sheriff M Pavlidis N. Aberrations of DNA repair pathways in prostate cancer: a cornerstone of precision oncology. Expert Opin Ther Targets. 2021;25:329–33. 34225539 10.1080/14728222.2021.1951226

[11] Castro E Romero-Laorden N Del Pozo A. PROREPAIR-B: a prospective cohort study of the impact of germline DNA repair mutations on the outcomes of patients with metastatic castration-resistant prostate cancer. J Clin Oncol. 2019;37:490–503. 30625039 10.1200/JCO.18.00358

[12] Ghose A Moschetta M Pappas-Gogos G Sheriff M Boussios S. Genetic aberrations of DNA repair pathways in prostate cancer: translation to the clinic. Int J Mol Sci . 2021;22:9783. 34575947 10.3390/ijms22189783 PMC8471942

[13] Hussain M Mateo J Fizazi K. PROfound Trial Investigators. Survival with olaparib in metastatic castration-resistant prostate cancer. N Engl J Med. 2020;383:2345–57. 32955174 10.1056/NEJMoa2022485

[14] Bourlon MT Valdez P Castro E. Development of PARP inhibitors in advanced prostate cancer. Ther Adv Med Oncol. 2024;16:17588359231221337. 38205078 10.1177/17588359231221337 PMC10777773

[15] Gandaglia G Abdollah F Schiffmann J. Distribution of metastatic sites in patients with prostate cancer: a population-based analysis. Prostate. 2014;74:210–6. 24132735 10.1002/pros.22742

[16] Krathen RA Orengo IF Rosen T. Cutaneous metastasis: a meta-analysis of data. South Med J. 2003;96:164–7. 12630642 10.1097/01.SMJ.0000053676.73249.E5

[17] Mueller TJ Wu H Greenberg RE. Cutaneous metastases from genitourinary malignancies. Urology. 2004;63:1021–6. 15183939 10.1016/j.urology.2004.01.014

[18] Sharma H Franklin M Braunberger R Hashemi-Sadraei N. Cutaneous metastasis from prostate cancer: a case report with literature review. Curr Problems Cancer: Case Rep. 2022;7:100175.

[19] Brown G Kurtzman D Lian F Sligh J. Eruptive nodules of the head and neck: a case report of metastatic prostate cancer. Dermatol Online J. 2014;20:doj_21544. 24612577

[20] Piqué Duran E Paradela A Fariña MC. Cutaneous metastases from prostatic carcinoma. J Surg Oncol. 1996;62:144–7. 8649042 10.1002/(SICI)1096-9098(199606)62:2<144::AID-JSO12>3.0.CO;2-8

[21] Reddy S Bang RH Contreras ME. Telangiectatic cutaneous metastasis from carcinoma of the prostate. Br J Dermatol. 2007;156:598–600. 17300266 10.1111/j.1365-2133.2006.07696.x

[22] Brown GT Patel V Lee CC. Cutaneous metastasis of prostate cancer: a case report and review of the literature with bioinformatics analysis of multiple healthcare delivery networks. J Cutan Pathol. 2014;41:524–8. 24456209 10.1111/cup.12296

[23] Rodríguez-Lojo R Castiñeiras I Rey-Sanjurjo JL Fernández-Díaz ML. Distant cutaneous metastases of prostate cancer: a report of 2 cases. Actas Dermosifiliogr. 2016;107:e52–6. 27085465 10.1016/j.ad.2015.09.021

[24] Rattanasirivilai A Kurban A Lenzy YM Yaar R. Cutaneous metastasis of prostatic adenocarcinoma: a cautionary tale. J Cutan Pathol. 2011;38:521–4. 21306410 10.1111/j.1600-0560.2011.01677.x

[25] Wang SQ Mecca PS Myskowski PL Slovin SF. Scrotal and penile papules and plaques as the initial manifestation of a cutaneous metastasis of adenocarcinoma of the prostate: case report and review of the literature. J Cutan Pathol. 2008;35:681–4. 18201228 10.1111/j.1600-0560.2007.00873.x

[26] Fukuda H Saito R. A case of Sister Mary Joseph’s nodule from prostatic cancer. J Dermatol. 2006;33:46–51. 16469085 10.1111/j.1346-8138.2006.00009.x

[27] Wu JJ Huang DB Pang KR Tyring SK. Cutaneous metastasis to the chest wall from prostate cancer. Int J Dermatol. 2006;45:946–8. 16911381 10.1111/j.1365-4632.2004.02503.x

[28] Oh TH Kim HS Park SC. Scalp nodules as the first presentation of prostate cancer: a CARE-compliant article. Medicine (Baltim). 2023;102:e36570. 10.1097/MD.0000000000036570 PMC10727530 38115333

[29] Esquivel-Pinto IA Torres-Alvarez B Gómez-Villa RJ Castanedo-Cázares JP. A case of prostatic carcinoma manifesting as cutaneous facial nodule. Case Rep Urol. 2018;2018:5265909. 29682391 10.1155/2018/5265909 PMC5846346

[30] Dills A Obi O Bustos K Jiang J Gupta S. Cutaneous metastasis of prostate adenocarcinoma: a rare presentation of a common disease. J Investig Med High Impact Case Rep. 2021;9:2324709621990769. 10.1177/2324709621990769 PMC7897805 33596692

[31] Alcaraz I Cerroni L Rutten A Kutzner H Requena L. Cutaneous metastases from internal malignancies: a clinicopathologic and immunohistochemical review. Am J Dermatopathol. 2012;34:347–93. 22617133 10.1097/DAD.0b013e31823069cf

[32] Garudadri G Rao BV Sundaram C. Diagnostic utility of immunohistochemical marker prostein for evaluation of primary and metastatic prostatic carcinomas. Indian J Pathol Microbiol. 2020;63:S18–24. 32108621 10.4103/IJPM.IJPM_852_18

[33] Wong JK Minni JP Nowak MA. A novel case of NKX3.1-positive metastatic cutaneous prostate cancer. Dermatol Online J. 2018;24:13030. 30677815

[34] Mannweiler S Amersdorfer P Trajanoski S Terrett JA King D Mehes G. Heterogeneity of prostate-specific membrane antigen (PSMA) expression in prostate carcinoma with distant metastasis. Pathol Oncol Res. 2009;15:167–72. 18802790 10.1007/s12253-008-9104-2

[35] Dall’Era MA True LD Siegel AF Porter MP Sherertz TM Liu AY. Differential expression of CD10 in prostate cancer and its clinical implication. BMC Urol. 2007;7:3. 17335564 10.1186/1471-2490-7-3 PMC1829163

[36] Li R Heydon K Hammond ME. Ki-67 staining index predicts distant metastasis and survival in locally advanced prostate cancer treated with radiotherapy: an analysis of patients in radiation therapy oncology group protocol 86-10. Clin Cancer Res. 2004;10:4118–24. 15217948 10.1158/1078-0432.CCR-1052-03

[37] Bailey C Broadbent A. Cutaneous metastases of prostate cancer. J Palliat Med. 2007;10:980–2. 17803422 10.1089/jpm.2007.9928

[38] Mak G Chin M Nahar N De Souza P. Cutaneous metastasis of prostate carcinoma treated with radiotherapy: a case presentation. BMC Res Notes. 2014;7:505. 25103825 10.1186/1756-0500-7-505 PMC4266901

[39] Sivrikoz IA Deveci H Ak AM Acer E. 68Ga-PSMA PET/CT images of multiple cutaneous metastases in a patient with prostate carcinoma: complete response to 177Lu-PSMA-617 treatment. Clin Nucl Med. 2023;48:950–2. 37812519 10.1097/RLU.0000000000004817

[40] Brito Y Wilson BW Bacchus KI Mwaniki J Jorge J Tiesenga F. Cutaneous metastases in progressive prostate adenocarcinoma: a case report and literature review. Cureus. 2023;15:46219. 10.7759/cureus.46219 PMC10613505 37905289

[41] Schoenlaub P Sarraux A Grosshans E Heid E Cribier B. Survie après métastases cutanées: étude de 200 cas [Survival after cutaneous metastasis: a study of 200 cases]. Ann Dermatol Venereol. 2001;128:1310–5. 11908133

[42] Marquis W Benson R. Long–term survival with skin metastases from carcinoma of prostate. Urology. 1980;16:407–8. 7414790 10.1016/0090-4295(80)90152-1

[43] Miyazaki T Kurosu M Hasikabe M Takahara M Yamakage A Yamazaki S. A case of skin metastasis of prostate cancer responding well to hormone therapy (in Japanese). Skin Cancer. 1999;14:259–64.

[44] Sarangi SS Singh V Bhirud DP. Cutaneous metastasis of castration-resistant prostate cancer: a rare case report and review of literature. Urol Ann. 2023;15:98–100. 37006211 10.4103/ua.ua_102_22 PMC10062501

[45] Oh SR Park JW Kwon HY Rha SH. BRCA1-mutated ovarian cancer with skin metastasis: a case report. Obstet Gynecol Sci. 2017;60:477–80. 28989926 10.5468/ogs.2017.60.5.477 PMC5621079

[46] Nie X Chen X Jiang Y Zhong Y Chen T Cheng W. Sister Mary Joseph nodule as cutaneous manifestations of metastatic ovarian cancer: a case report and review of the literature. Medicine (Baltim). 2022;101:e28712. 10.1097/MD.0000000000028712 PMC8830813 35147092

[47] Caputo R Pagliuca M Pensabene M Parola S De Laurentiis M. Long-term complete response with third-line PARP inhibitor after immunotherapy in a patient with triple-negative breast cancer: a case report. Front Oncol. 2023;13:1214660. 37601649 10.3389/fonc.2023.1214660 PMC10438988

[48] Minick TB Norman RA. Somatic BRCA mutation in metastatic breast cancer. Cureus. 2023;15:e49600. 38161892 10.7759/cureus.49600 PMC10755085

[49] Assaf I Mans L Sakr R Verset G Van Laethem JL. Unusual metastasis in BRCA mutated pancreatic cancer while on maintenance Olaparib: two case reports and review of the literature. Eur J Cancer. 2021;157:63–7. 34487986 10.1016/j.ejca.2021.07.042

